# Papel dos Níveis de Sódio na Fibrilação Atrial na Insuficiência Cardíaca: Jogador Ativo ou Bystander?

**DOI:** 10.36660/abc.20210139

**Published:** 2022-01-11

**Authors:** Aydın Akyüz, Derya Baykız, Demet Özkaramanlı Gür, Sümeyra Gökçek, Muhammet Mucip Efe, Şeref Alpsoy

**Affiliations:** 1 Namik Kemal University Faculty of Medicine Tekirdag Turquia Namik Kemal University Faculty of Medicine – Cardiology, Tekirdag – Turquia; 2 Istanbul University Istanbul Faculty of Medicine Istanbul Turquia Istanbul University Istanbul Faculty of Medicine – Cardiology, Istanbul – Turquia

**Keywords:** Hiponatremia, Fibrilação Atrial, Insuficiência Cardíaca

## Abstract

**Fundamento:**

A coexistência de hiponatremia e fibrilação atrial (FA) aumenta a morbidade e mortalidade em pacientes com insuficiência cardíaca (IC). No entanto, não está estabelecido se a hiponatremia está relacionada à FA ou não.

**Objetivo:**

O objetivo do nosso estudo foi buscar a possível associação de hiponatremia com FA em pacientes que apresentam IC com fração de ejeção reduzida (ICFrE).

**Métodos:**

Este estudo observacional, transversal e unicêntrico incluiu 280 pacientes ambulatoriais consecutivos com diagnóstico de ICFr com 40% ou menos. Com base nas concentrações de sódio ≤135 mEq/L ou superior, os pacientes foram classificados em hiponatremia (n=66) e normonatremia (n=214). Um valor de p<0,05 foi considerado significativo.

**Resultados:**

A média de idade foi de 67,6±10,5 anos, 202 (72,2%) eram do sexo masculino, o nível médio de sódio no sangue foi de 138±3,6 mEq/L e a fração de ejeção média foi de 30±4%. Ao todo, 195 (69,6%) pacientes foram diagnosticados com doença arterial coronariana. A FA foi detectada em 124 (44.3%) pacientes. A taxa de FA foi maior em pacientes com hiponatremia em comparação com aqueles com normonatremia (n=39 [59,1%] vs. n=85 [39,7%), p=0,020). Na análise de regressão logística, a hiponatremia não foi relacionada à FA (OR=1.022, IC 95%=0,785–1.330, p=0,871). Idade aumentada (OR=1.046, IC 95%=1.016–1.177, p=0,003), presença de DAC (OR=2.058, IC 95%=1,122–3.777, p=0,020), frequência cardíaca em repouso (OR=1.041, IC 95%=1.023–1.060, p<0,001) e diâmetro do átrio esquerdo (OR=1.049, IC 95%=1.011–1.616, p=0,002) foram considerados preditores de FA.

**Conclusão:**

A FA foi uma taxa mais elevada em pacientes ambulatoriais com ICFr e hiponatremia. No entanto, não há associação entre os níveis de sódio e FA em pacientes com ICFrEF.

## Introdução

A insuficiência cardíaca (IC) é categorizada com base na fração de ejeção (FE) como FE reduzida ≤0,40 (ICrFE), FE preservada ≥0,50 (ICpFE) ou FE média (<0,50, mas >0,40). Sua taxa vem aumentando gradativamente, estando relacionada a altas taxas de hospitalização e mortalidade.^1 , 2^

Anemia, infecção, isquemia miocárdica, insuficiência renal, fibrilação atrial (FA) e anormalidades eletrolíticas são fatores predisponentes comuns para o agravamento da IC e podem contribuir para o desenvolvimento de sintomas clínicos de IC, como dispneia, fadiga e edema ou atividade limitada.

Embora as alterações no sódio, pelo menos teoricamente, possam contribuir para o risco de arritmia, sabe-se que distúrbios do equilíbrio eletrolítico de potássio, cálcio e magnésio desencadeiam arritmias. Define-se hiponatremia como concentração sérica de sódio ≤135 mEq/L, uma das anormalidades eletrolíticas mais comuns, associada a resultados desfavoráveis em pacientes com IC com prevalência de cerca de 13,8%–33,7%.^3 - 5^

A prevalência de FA em pacientes com ICrFE varia de <10% a 50%.^5 - 7^ A FA na IC é uma arritmia incapacitante comum associada à gravidade da doença, alta morbidade e mortalidade. A FA leva à IC e vice-versa.^1 , 8 - 10^ Embora a relação entre FA e o desequilíbrio eletrolítico seja teoricamente bem conhecida, a associação de hiponatremia com o desenvolvimento de FA na IC não está bem documentada na literatura. Pela primeira vez, uma associação causal entre hiponatremia e desenvolvimento de FA foi descrita em um estudo recente de Cavusoglu et al.^11^ Algum ceticismo, entretanto, ainda existe sobre o papel da baixa concentração de sódio no desenvolvimento de FA na IC, o que demonstrou a necessidade de mais estudos.^12^

Considerando essa possível relação, objetivamos investigar se há uma associação independente ou predisposição recíproca entre hiponatremia e FA em nossos pacientes com ICrFE.

## Métodos

Neste estudo transversal, pacientes nas classes funcionais I–IV da New York Heart Association (NYHA) em atendimento ambulatorial com diagnóstico de IC sistólica crônica com fração de ejeção (FE) de 40% ou menos foram recrutados consecutivamente. O protocolo do estudo foi aprovado pelo comitê de ética local (2019.152.09.12). Todos os indivíduos forneceram consentimento informado por escrito antes de se inscreverem no estudo.

Pacientes com menos de 18 anos, pacientes com doença cardíaca congênita, doença valvar moderada a grave, miocardite ativa, síndromes coronarianas agudas nos últimos 3 meses, doenças inflamatórias, neoplasias, doença renal crônica ou hepática grave com taxa de filtração glomerular estimada (eTFG) ≤30 mL/min, cardiomiopatia hipertrófica, distúrbios da tireoide, doença pulmonar obstrutiva crônica, anemia grave e aqueles com ICpFE ou sintomas agudamente descompensados de classe IV da NYHA que necessitariam de suporte inotrópico no mês anterior foram excluídos.

Os pacientes foram divididos em 2 grupos com base nos níveis de sódio (≤135 mEq/L e >135 mEq/L: hiponatremia e normonatremia. O estudo utilizou 280 pacientes (202 homens e 78 mulheres). Realizou-se análise de potência de acordo com a comparação dos grupos hiponatremia e normonatremia na presença de FA. A potência do estudo foi de 83,7% com 95% de confiabilidade. Portanto, o tamanho da amostra do estudo foi adequado para validar os resultados.

Idade, sexo, tabagismo atual, presença de diabetes mellitus (DM), hipertensão (HT) ou hiperlipidemia (HL), medicamentos utilizados e tempo de doença foram registrados para todos os indivíduos na primeira consulta médica. Utilizou-se eletrocardiograma (ECG) de 12 derivações feito em repouso para determinar a frequência cardíaca de repouso e o ritmo sinusal ou fibrilação atrial. Todos os pacientes com ritmo sinusal normal no ECG de repouso foram investigados por um registrador de ECG ambulatorial de três canais por 24 horas (MT-200, Schiller AG, Baar, Suíça) para descartar FA paroxística.

Todos os pacientes foram submetidos a ecocardiografia transtorácica detalhada (GE Vingmed Ultrasound AS, Horten, Noruega) como parte do protocolo do estudo. Utilizou-se o método de Simpson modificado para calcular a FE ventricular esquerda. Foram medidos os diâmetros diastólico ventricular esquerdo (VE) e sistólico atrial esquerdo (AE). As velocidades de regurgitação tricúspide foram determinadas por ecocardiografia Doppler de onda contínua e a pressão sistólica da artéria pulmonar (PSAP) foi calculada de acordo com as recomendações das diretrizes atuais.^13^

Estabeleceu-se o diagnóstico de hipertensão (HT) como pressão sistólica ≥140 mm Hg e/ou pressão diastólica ≥90 mm Hg em mais de duas ocasiões ou uso de qualquer medicação anti-hipertensiva. Diagnosticou-se DM como glicemia de jejum superior a 126 mg/dL ou uso de medicamentos antidiabéticos. Definiu-se doença arterial coronariana (DAC) com base em angiografia coronária como estreitamento do diâmetro ≥50% em artéria coronária epicárdica.

Amostras de sangue venoso em jejum foram coletadas na parte da manhã para determinar glicose de jejum, creatinina, colesterol de lipoproteína de baixa densidade (LDL), ácido úrico, sódio, potássio, proteína C-reativa ultrassensível (PCRus) e níveis de hemoglobina. Calculou-se a osmolalidade sérica (miliosmoles por quilograma) como (2 × Na) + (BUN/2.8) + (glucose/18), conforme descrito anteriormente.^14^

Mediu-se a concentração sérica da porção N-terminal do pró-hormônio do peptídeo natriurético tipo B (NT-pro-BNP) pelo imunoensaio tipo sanduíche Elecsys proBNP (Elecsys 2010, Roche Diagnostics). A faixa analítica variou entre 5 a 35000 pg/mL. Os coeficientes de variação (CV) interensaio e intraensaio de NT-proBNP nas faixas baixa e alta foram relatados como 8,8%–11,6% e 9,9–12,2%, respectivamente. O kit de PCRus humano (kit ELISA de proteína C-reativa de alta sensibilidade, DRG International Inc, NJ, EUA) incluiu CV% interensaio e intraensaio <4,1% e <7,5%; a dose mínima detectável de PCR-us foi de 0,01 mg/L.

### Análise estatística

A análise estatística foi realizada por meio do software preditivo Analysis Software Statistics 18 (SPSS Inch, Chicago, Illinois, EUA). As variáveis foram testadas para verificar a normalidade da distribuição pelo teste de Kolmogorov-Smirnov. As variáveis com distribuição normal foram apresentadas como média±desvio padrão (DP), as sem distribuição normal foram apresentadas como mediana e intervalo interquartil. Dois testes t de amostra independente foram usados para comparar dados normalmente distribuídos e o teste U de Mann-Whitney foi usado para comparar dados não normalmente distribuídos. As variáveis categóricas foram apresentadas em números (porcentagem). As comparações entre as variáveis categóricas dos dois grupos foram feitas pelo teste do qui-quadrado. Realizamos análises de regressão logística univariada e multivariada para avaliar os preditores de FA. Para a análise multivariada, as variáveis com valores de p <0,1 foram inseridas no modelo por um método *forward stepwise* . Para verificar o melhor ponto de sensibilidade e especificidade do valor de corte de sódio para a previsão de FA, utilizou-se a análise da curva ROC ( *Receiver Operator Characteristic* ). Considerou-se significativo um p bicaudal <0,05.

## Results

De 376 pacientes ambulatoriais consecutivos com diagnóstico de IC, 96 com características que satisfaziam os critérios de exclusão não foram incluídos no estudo. Os motivos de exclusão foram síndrome coronariana aguda em 20, doença pulmonar obstrutiva crônica em 10, eTFG ≤30 mL/min em 49, distúrbios inflamatórios em 17 pacientes e nenhuma angiografia coronária anterior para definir a etiologia em oito pacientes. Portanto, o tamanho da amostra foi composto por pacientes classificados em dois grupos de acordo com suas concentrações de sódio, da seguinte forma: o grupo hiponatremia tinha 66 pacientes e o grupo normonatremia tinha 214 pacientes.

Os dados demográficos e as características da população do estudo são apresentados na Tabela 1 . Na população geral do estudo, a média de idade foi de 67,6±10,5 anos; o nível médio de sódio no sangue foi de 138±3,6 mEq/L, e o número (%) de pacientes com FA foi 124 (44,3%). Dos pacientes com FA, 96 pacientes tinham FA permanente, enquanto 28 pacientes (22,5%) tinham FA paroxística. Os níveis de sódio no grupo hiponatremia e no grupo normonatremia foram 132±3,7 e 140±2,7 mEq/L, respectivamente.

**Tabela 1:** – Características clínicas, variáveis laboratoriais e ecocardiográficas, e medicamentos

Variáveis	Todos os pacientes n=280	Hiponatremia Grupo n=66	Grupo normonatremia n=214	Valor de p
Idade, anos	67.6±10,5	67±11	68±10	0,820
Sexo masculino, n (%)	202 (72,2)	47 (71,2)	155 (72,4)	0,847
Hipertensão, n (%)	185 (66,1)	41 (62,1)	144 (67,3)	0,438
Diabetes mellitus, n (%)	96 (34,3)	32 (48,5)	64 (29,9)	0,005
Doença arterial coronariana, n (%)	195 (69,6)	44 (66,7)	151 (70,6)	0,548
Fibrilação atrial, n (%)	124 (44,3)	39 (59,1)	85 (39,7)	0,020
NYHA classe I–II n (%)	176 (62,9)	45 (68,2)	131 (61,2)	0,306
NYHA classe I–II +AF, n (%)		23/45 (51,1) ^a^	42/131 (32,1) ^c^	0,022
NYHA classe III–IV, n (%)	104 (37,1)	21 (31,8)	83 (38,8)	0,306
NYHA classe III–IV +AF, n (%)		15/21 (71,4) ^b^	44/83 (53%) ^d^	0,028
Duração da doença (anos)	5,5 (3–12)	5,1 (4–11)	5,4 (3–9)	0,546
Frequência cardíaca em repouso (bpm)	82,5 ±19	82±12	84±19	0,215
**Exames laboratoriais**				
Glicemia de jejum (mg/dL)	125±55	136±61	121±52	0,041
Creatinina (mg/dL)	124±0,3	1.25±0,30	1.24±0,3	0,662
Colesterol LDL (mg/dL)	103±42	106±49	102±40	0,461
Ácido úrico (mg/dL)	7.4±2,4	7.6±2,4	7.3±2,3	0,294
Sódio (mEq/L)	138±3,6	132±3,7	140±2,7	<0,001
Potássio (mEq/L)	4.4±0,5	4.5±0,5	4.3±0,5	0,513
PCRus (mg/dL)	3,8 (1,5–7,3)	4,2 (1,8–6,7)	3,6 (1,2–7,8)	0,367
NT-ProBNP, pg/mL	2605 (903–6825)	2916 (1170–9566)	2378 (867–6015)	0,199
Osmolalidade (mOsm/kg)	291±9	283±9	294±7	<0,001
Hemoglobina (g/dL)	12.8±2	12.5±1,7	12.9±1,9	0,393
**Parâmetros ecocardiográficos**				
Diâmetro AE (mm)	46±7	45±6	46±7	0,546
Diâmetro diastólico do VE (mm)	59±7	59±7	60±8	0,634
Fração de ejeção (%)	30±4	29±4	31±4	0,518
PSAP (mmHg)	42±14	40±13	42±14	0,343
Medicações				
IECA/BRA n (%)	184 (65,7)	38 (57,6)	146 (68,2)	0,070
ARM, n (%)	157 (56,1)	44 (66,7)	113 (52,8)	0,021
Diuréticos, n (%)	208 (74,3)	48 (72,7)	160 (74,8)	0,194
Betabloqueadores, n (%)	236 (84,3)	54 (81,8)	182 (85)	0,183
Digoxina, n (%)	56 (20)	19 (28,8)	37 (17,3)	0,022

*IECA: Inibidor da enzima conversora da angiotensina; BRA: Bloqueador do receptor de angiotensina; FA: Fibrilação atrial; PCRus: Proteína C-reativa de alta sensibilidade; AE: Átrio esquerdo; LDL: Lipoproteína de baixa densidade, VE: Ventrículo esquerdo; mOsm/kg: Miliosmoles por quilograma; ARM: Antagonista dos receptores de mineralocorticoides; NT-proBNP: porção N-terminal do pró-hormônio do peptídeo natriurético tipo B; PSAP: Pressão sistólica da artéria pulmonar. Entre a e b, p=0120; entre c e d, p=0,002.*

**Figura 1:**
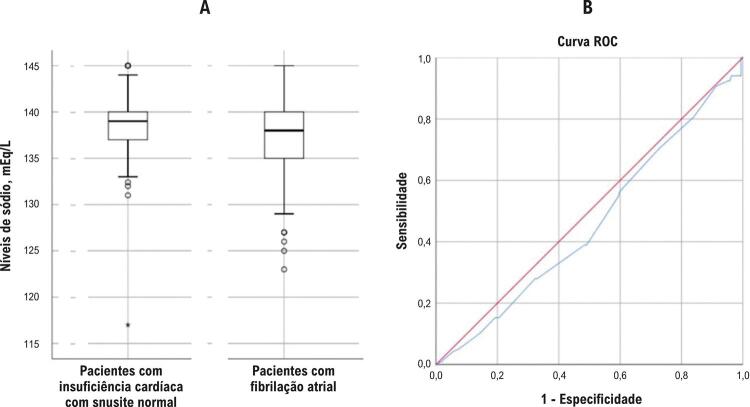
– A) Mostra a comparação dos níveis de sódio entre os pacientes que apresentam insuficiência cardíaca com sinusite normal e fibrilação atrial. B) Demonstra a análise ROC que mostra uma sensibilidade diagnóstica pobre e especificidade dos níveis de sódio para prever a possibilidade.

O grupo com hiponatremia apresentou maior proporção de FA e DM do que o grupo com normonatremia. As proporções de pacientes com hipertensão, DAC, diabetes mellitus e classe funcional III–IV da NYHA foram semelhantes nos dois grupos. A glicose de jejum, as taxas de antagonista dos receptores de mineralocorticoides (ARM) e o uso de digoxina foram maiores no grupo com hiponatremia em comparação com o grupo com normonatremia. A osmolalidade foi menor no grupo com hiponatremia, conforme naturalmente esperado. Idade, sexo, duração da doença, frequência cardíaca em repouso, creatinina, colesterol LDL, ácido úrico, potássio, PCRus, NT-proBNP, hemoglobina, diâmetro diastólico LA e VE, FE (%) e valores de PSAP foram semelhantes nos dois grupos. Pacientes com FA apresentaram menores níveis de sódio em comparação com aqueles sem FA (136±4,3 vs. 138±3,0 mEg/L, p=0,001) (Figura 1A) ( Tabela 1 ).

Em pacientes com hiponatremia, as taxas de FA foram significativamente maiores em pacientes em classes funcionais NYHA mais elevadas. Embora não tenha havido diferença em termos de taxas de FA entre as classes I–II e III–IV da NYHA em pacientes com hiponatremia e ICrFE, as taxas de FA mostraram diferença estatisticamente significativa em pacientes com normonatremia e IC ( Tabela 1 ).

Os resultados da análise de regressão logística univariada e multivariada para preditores independentes de FA revelaram idade avançada, frequência cardíaca de repouso e diâmetro do AE. O uso de diurético e digoxina apresentou forte correlação com a presença de FA ( Tabela 2 ).

**Tabela 2:** – Análises de regressão logística univariada e multivariada para a presença de fibrilação atrial

		Razão de chances	IC 95%	p
**Análises univariadas**				
Idade	0,076±0,023	1,079	1,031–1.170	0,001
Sexo masculino	0,652±0,270	1,919	1,131–3.256	0,016
Hipertensão	0,336±0,432	1,399	0,600–3.260	0,437
Diabetes mellitus	0,246±0,487	1,279	0,492–3.325	0,613
Doença arterial coronariana	-0,805±0,451	0,447	0,185–1.081	0,074
Capacidade funcional	0,026±0,419	1,027	0,451–2.334	0,950
Duração da doença	1,196±0,576	0,827	0,271–2.493	0,729
Frequência cardíaca em repouso	0,041±0,008	1,042	1,026–1.059	<0,001
Glicemia de jejum	0,001±0,005	1,001	0,990–1.011	0,924
Creatinina	-0,025±0,182	0,976	0,682–1.395	0,892
Colesterol LDL	-0,007±0,003	0,993	0,987–0.999	0,024
Ácido úrico	0,172±0,100	1,188	0,976–1.446	0,086
Níveis de sódio	0,022±0,134	1,022	0,785–1.330	0,871
Potássio	-0,727±0,391	0,483	0,225–1.039	0,063
PCRus	-0,012±0,016	0,988	0,958–1.020	0,461
NT-ProBNP	0,001±0,001	1,000	0,999–1.001	0,071
Osmolalidade	-0,065±0,060	0,937	0,834–1.054	0,279
Hemoglobina	-0,174±0,131	0,840	0,650–1.086	0,183
Diâmetro do AE	0,046±0,013	1,047	1,021–1.516	<0,001
IECA/BRA	-0,047±0,288	0,954	0,543–1.677	0,870
ARM	-0,163±0,290	0,850	0,481–1.501	0,575
Diuréticos	1,448±0,364	4,256	2,086–8.685	<0,001
Betabloqueadores	-0,165±0,388	0,848	0,396–1.814	0,671
Digoxina	1,876±0,365	6,526	3,193–13.340	<0,001
**Análise multivariada**				
Idade	0,045±0,015	1,046	1,016–1.177	0,003
Doença arterial coronariana	-0,805±0,451	2,058	1,122–3.777	0,020
Frequência cardíaca em repouso	0,041±0,009	1,041	1,023–1.060	<0,001
Diâmetro do AE	0,044±0,017	1,049	1,011–1.616	<0,001

*IECA: Inibidor da enzima conversora da angiotensina; BRA: Bloqueador do receptor de angiotensina; PCRus: Proteína C-reativa ultrassensível; AE: Átrio esquerdo; LDL: Lipoproteína de baixa densidade; NT-proBNP: Porção N-terminal do pró-hormônio do peptídeo natriurético tipo B; ARM: Antagonista dos receptores de mineralocorticoides.*

A análise ROC (AUC=0,458, IC 95%=0,397–0,527) revelou que os níveis de sódio no sangue ≤135 mEq/L têm baixa sensibilidade (55%) e especificidade (41%) diagnóstica para prever FA. Com o valor de corte do nível de sódio ajustado para ≤130 mEq/L, encontravam-se valores de maior sensibilidade (70%) e baixa especificidade (31%) (Figura 1B).

## Discussão

Relatamos uma prevalência de FA maior em pacientes ambulatoriais com ICrFE e hiponatremia do que naqueles com ICrFE e normonatremia, independentemente dos níveis de osmolalidade plasmática e outros fatores de confusão. Há dois estudos na literatura que mostram taxas mais elevadas de FA em pacientes com ICrFE e hiponatremia,^5 , 11^ com os quais nossos resultados são concordantes. No entanto, a hiponatremia não foi um fator estimulante para o desenvolvimento de FA no estudo. A hiponatremia é sensível, de leve a moderada, mas não é específica para predizer o desenvolvimento de FA. Ou seja, a FA não está presente em todos os pacientes com hiponatremia e ICrFE.

Os fatores predisponentes e determinantes importantes para o desenvolvimento de FA foram idade avançada, presença de DAC, aumento da frequência cardíaca em repouso e dimensão do AE, estabelecidos como preditores de FA por estudos prévios.^9 , 10 , 15^ Relatamos uma taxa de FA mais alta em pacientes com hiponatremia, independentemente de sua classe funcional NHYA, conforme documentado anteriormente.^5 , 11^ Portanto, a coexistência de hiponatremia e FA pode demonstrar gravidade da IC. As taxas de FA nos pacientes com normonatremia e IC foram maiores nas classes NHYA III–IV, o que também significa que a presença da classe NYHA III–IV é um motivo importante para o uso de diuréticos. Portanto, a hiponatremia parece ser apenas uma variável *bystander* .

Os motivos mais comuns de hiponatremia em pacientes com IC são o uso de diuréticos e a resposta neuro-hormonal, incluindo um desequilíbrio autonômico em favor do sistema nervoso simpático ou ativação do sistema renina-angiotensina (SRA).^1 , 5 , 16^

Muitos fatores têm sido responsáveis pela associação de hiponatremia com aumento do risco de FA. A insuficiência cardíaca reduz o volume sistólico e o enchimento arterial, resultando na estimulação dos barorreceptores arteriais, liberação de arginina vasopressina (AVP) e ativação de SRA. A ativação do SRA leva ao aumento dos níveis de aldosterona e angiotensina II. A angiotensina II alerta o centro de sede do cérebro e estimula a liberação de AVP. O aumento subsequente dos níveis de aldosterona, angiotensina II, sistema simpático e liberação de AVP induz a redução do fluxo sanguíneo renal, aumento da retenção de água e reabsorção de sódio.^4 , 17^ Como resultado dessas alterações neuro-hormonais, ocorrem hipervolemia e hiponatremia. Alguns estudos mostraram níveis aumentados de renina, angiotensina II, aldosterona, epinefrina, norepinefrina e dopamina em pacientes com IC e hiponatremia em comparação com aqueles com IC e normonatremia.^16 - 19^

A hiponatremia também pode ser preditor de maior ativação neuro-hormonal que sugere gravidade da IC.^4^ Diuréticos, principalmente tiazidas, muitas vezes resultam em hiponatremia, que promove retenção de água devido ao aumento da ativação de AVP nos túbulos distais.^20 , 21^ A hipervolemia leva não apenas à hiponatremia mas também ao estiramento do miocárdio atrial, câmara cardíaca e dilatação da veia pulmonar.^22^ A hiponatremia, teoricamente, também pode contribuir para o desenvolvimento de FA, causando alterações eletrofisiológicas no potencial de ação do miócito.^23^ No entanto, na prática clínica, parece não ser um determinante da FA.

A frequência cardíaca rápida induzida pela FA afeta negativamente a função do ventrículo esquerdo, facilita a taquicardia e predispõe à apoptose e à fibrose miocárdica. Independentemente da presença de IC, a frequência cardíaca irregular e a perda da contração atrial resultam em redução significativa de 7–9% e 20% no débito cardíaco, respectivamente.^24^ Quando a IC e a FA coexistem, duas entidades interrelacionadas fazem o débito cardíaco diminuir sinergicamente, aumentando a mortalidade.^8^ Há uma relação de causa-efeito comum entre essas duas entidades.

A hiponatremia é frequentemente observada em pacientes com IC descompensada aguda devido ao alto uso de diuréticos e alto tônus simpático desencadeando a ativação do SRA.^5 , 21^ Nossos achados não são concordantes com o estudo de Cavusoglu et al., que apresentou hiponatremia com prevalência de 24% e FA com prevalência de 33%.^11^ Bavishi et al. verificaram que a prevalência de hiponatremia e FA em pacientes ambulatoriais com ICrFE foi de 14,8% e 37,6%, respectivamente.^5^ Nosso estudo apresentou uma taxa de hiponatremia de 23,5%, mas uma taxa de FA maior, de 44,3%, porque realizamos ECG Holter ambulatorial para verificar a presença de FA paroxística ou persistente. As taxas de FA são mais altas do que esperávamos no monitoramento ambulatorial Holter ECG de 24 horas em pacientes com IC.^2 , 15^

### Limitações do estudo

Apresentamos dados ausentes relacionados às doses de diuréticos e níveis de albumina que podem afetar os níveis de sódio.

## Conclusão

Os achados atuais fornecem informações sobre a patogênese da FA em pacientes com IC estabelecida. Embora a hiponatremia desempenhe um papel fundamental na deterioração do estado da IC, verificamos que a baixa concentração de sódio sérico, ≤135 mEq/L, não está relacionada à probabilidade de FA.
