# Supplementary material for: Papel dos Níveis de Sódio na Fibrilação Atrial na Insuficiência Cardíaca: Jogador Ativo ou Bystander?
Source: Arq Bras Cardiol. 2022 Jan 11;118(4):712–8. [Article in Portuguese] doi: 10.36660/abc.20210139 (PMC9007004; doi:10.36660/abc.20210139)
Supplement: Translation

# Translation: Role of Sodium Levels on Atrial Fibrillation in Heart Failure: Active Player or a Bystander?

**Authors:** Aydın Akyüz, Derya Baykız, Demet Özkaramanlı Gür, Sümeyra Gökçek, Muhammet Mucip Efe, Şeref Alpsoy

**Affiliations:** 1 Turkey Namik Kemal University Faculty of Medicine – Cardiology, Tekirdag – Turkey; 2 Turkey Istanbul University Istanbul Faculty of Medicine – Cardiology, Istanbul – Turkey

**Keywords:** Hyponatremia, Atrial Fibrillation, Heart Failure

## Abstract

**Background:**

The coexistence of hyponatremia and atrial fibrillation (AF) increases morbidity and mortality in patients with heart failure (HF). However, it is not established whether hyponatremia is related to AF or not.

**Objective:**

Our study aims to seek a potential association of hyponatremia with AF in patients with reduced ejection fraction heart failure (HFrEF).

**Methods:**

This observational cross-sectional single-center study included 280 consecutive outpatients diagnosed with HFrEF with 40% or less. Based on sodium concentrations ≤135 mEq/L or higher, the patients were classified into hyponatremia (n=66) and normonatremia (n=214). A p-value <0.05 was considered significant.

**Results:**

Mean age was 67.6±10.5 years, 202 of them (72.2%) were male, mean blood sodium level was 138±3.6 mEq/L, and mean ejection fraction was 30±4%. Of those, 195 (69.6%) patients were diagnosed with coronary artery disease. AF was detected in 124 (44.3%) patients. AF rate was higher in patients with hyponatremia compared to those with normonatremia (n=39 [59.1%] vs. n=85 [39.7%), p= 0.020). In the logistic regression analysis, hyponatremia was not related to AF (OR=1.022, 95% CI=0.785–1.330, p=0.871). Advanced age (OR=1.046, 95% CI=1.016–1.177, p=0.003), presence of CAD (OR=2.058, 95% CI=1.122–3.777, p=0.020), resting heart rate (OR=1.041, 95% CI=1.023–1.060, p<0.001), and left atrium diameter (OR=1.049, 95% CI=1.011–1.616, p=0.002) were found to be predictors of AF.

**Conclusion:**

AF was higher in outpatients with HFrEF and hyponatremia. However, there is no association between sodium levels and AF in patients with HFrEF.

## Introduction

Heart failure (HF) is categorized based on ejection fraction (EF) as reduced EF≤0.40 (HFrEF), preserved EF≥ 0.50 (HFpEF), or midrange EF (<0.50 but >0.40). Its rate is gradually increasing and is related to high rates of hospitalization and mortality.^1 , 2^

Anemia, infection, myocardial ischemia, renal failure, atrial fibrillation (AF), and electrolyte abnormalities are common predisposing factors for HF worsening and may contribute to the development of clinical symptoms of HF such as dyspnea, fatigue, and edema or limited activity.

Although sodium abnormalities, at least theoretically, may contribute to the risk of arrhythmia, disorders of electrolyte balance in potassium, calcium, and magnesium are well-known to have triggered arrhythmias. Hyponatremia is defined as serum sodium concentration ≤135 mEq/L, one of the most common electrolyte abnormalities, associated with poor outcome in patients with HF with a prevalence of about 13.8%–33.7%.^3 - 5^

The prevalence of AF in patients with HFrEF ranges from <10% to 50%.^5 - 7^ AF in HF is a common disabling arrhythmia associated with severity of disease, high morbidity, and mortality. AF leads to HF and vice versa.^1 , 8 - 10^ Although the relationship between AF and electrolyte imbalance is theoretically well-known, the association of hyponatremia with AF development in HF has not been well-documented in the literature. For the first time, a causative association between hyponatremia and AF development was claimed in a recent study by Cavusoglu et al.^11^ Some skepticism, however, still exists about the role of low sodium concentration on AF development in HF, which demonstrated the need for further studies.^12^

Considering this potential relationship, we aimed to investigate whether there is an independent association or reciprocal predisposition between hyponatremia and of AF in our patients with HFrEF.

## Methods

In this cross-sectional study, patients under the New York Heart Association (NYHA) functional classes I–IV admitted to the outpatient clinic with diagnosis of chronic systolic HF with ejection fraction (EF) of 40% or less were consecutively recruited. The study protocol was approved by the local Ethics committee (2019.152.09.12). All of the subjects provided written informed consent before enrolling in the study.

Patients younger than 18 years, patients with congenital heart disease, moderate to severe valvular disease, active myocarditis, acute coronary syndromes within the last 3 months, inflammatory disorders, malignancies, severe hepatic or chronic kidney disease with an estimated glomerular filtration rate (eGFR) ≤30 mL/min, hypertrophic cardiomyopathy, thyroid disorders, chronic obstructive pulmonary disease, severe anemia, and those with HFpEF or acutely decompensated symptoms showing NYHA class IV who would require inotropic support within the previous month were excluded.

Patients were divided into 2 groups based on sodium levels (≤135 mEq/L and >135 mEq/L: hyponatremia, and normonatremia. The study used 280 patients (202 males and 78 females). Power analysis was performed according to the comparison of hyponatremia and normonatremia groups in the presence of AF. The power of the study was 83.7% with 95% reliability. Therefore, the study sample size was suitable to validate the results.

Age, gender, current smoking status, presence of diabetes mellitus (DM), hypertension (HT), or hyperlipidemia (HL), medications used and disease duration were recorded for all subjects at the first medical consultation. A 12-lead resting electrocardiogram (ECG) was used to determine resting heart rate and sinus rhythm or atrial fibrillation. All patients with normal sinus rhythm in resting ECG were investigated using a 24-hour three-channel ambulatory ECG recorder (MT-200, Schiller A.G, Baar, Switzerland) to rule out paroxysmal AF.

All patients underwent detailed transthoracic echocardiography (GE Vingmed Ultrasound AS, Horten, Norway) as part of the study protocol. The modified Simpson method was used to calculate left ventricular EF. Left ventricle diastolic (LV) and left atrium systolic (LA) diameters were measured. Tricuspid regurgitation velocities were determined by continuous-wave Doppler echocardiography, and systolic pulmonary artery pressure (sPAP) was calculated according to the recommendations of current guidelines.^13^

Diagnosis of hypertension (HT) was established as systolic pressure ≥140 mm Hg and/or diastolic pressure ≥90 mm Hg on more than two occasions or use of any antihypertensive medication. DM was diagnosed as fasting blood glucose higher than 126 mg/dL or being on antidiabetic medication. Coronary artery disease (CAD) was defined based on a coronary angiogram as diameter narrowing ≥50% in an epicardial coronary artery.

Fasting venous blood samples were collected in the morning hours to determine fasting glucose, creatinine, low-density lipoprotein (LDL) cholesterol, uric acid, sodium, potassium, high-sensitivity C-reactive protein (hs-CRP), and hemoglobin levels. Serum osmolality (milliosmoles per kilogram) was calculated as (2 × Na) + (BUN/2.8) + (glucose/18) as described previously.^14^

Serum N terminal pro B-type natriuretic peptide (NT-pro-BNP) concentration was measured using Elecsys proBNP sandwich immunoassay (Elecsys 2010, Roche Diagnostics). The analytical range was between 5 to 35000 pg/mL. Interassay and intraassay coefficients of variation (CV) of NT-proBNP in the low and high ranges were reported as 8.8%–11.6% and 9.9–12.2%, respectively. Human hsCRP kit (High-Sensitivity C-Reactive Protein ELISA kit, DRG International Inc, NJ, USA) included inter-assay and intra-assay CV% <4.1% and <7.5%; the minimum detectable dose of hs-CRP was 0.01 mg/L.

### Statistical analysis

Statistical analysis was performed using the predictive Analysis Software Statistics 18 (SPSS Inch, Chicago, Illinois, USA). The variables were tested to check the normality of distribution by the Kolmogorov–Smirnov test. Variables with normal distributions were presented as mean±standard deviation (SD), those without normal distributions were presented as median and interquartile range. Two independent sample t-tests were used to compare normally distributed data and the Mann-Whitney U test was used to compare non-normally distributed data. The categorical variables were presented as numbers (percentage). Comparisons between the categorical variables of the two groups were made by chi-square test. We performed logistic univariate and multivariate regression analyses to assess the predictors of AF. For the multivariate analysis, variables with p values <0.1 were entered into the model by a forward stepwise method. To verify the best cut-off sodium value point of sensitivity and specificity for the prediction of AF, receiver operator characteristic (ROC) curve analysis was used. A two-tailed p<0.05 was considered significant.

## Results

Of 376 consecutive outpatients diagnosed with HF, 96 with characteristics satisfying the exclusion criteria were not included in the study. The reasons for exclusion were acute coronary syndrome in 20, chronic obstructive pulmonary disease in 10, eGFR ≤30 mL/min in 49, inflammatory disorders in 17 patients, and no prior coronary angiography to define the etiology in eight patients. Therefore, the sample size consisted of patients classified into two groups according to their sodium concentrations, as follows: the hyponatremia group included 66 patients, and the normonatremia group included 214 patients.

Demographic data and characteristics of the study population are presented in Table 1 . In the overall study population, the mean age was 67.6±10.5 years; mean blood sodium level was 138±3.6 mEq/L, and the number (%) of patients with AF was 124 (44.3%). Of patients with AF, 96 patients had permanent AF while 28 patients (22.5%) were determined to have paroxysmal AF. Sodium levels in the hyponatremia group and in the normonatremia group were 132±3.7 and 140±2.7 mEq/L, respectively.

**Table 1:** – Clinical characteristics, laboratory and echocardiographic variables, and medications

Variables	All Patients n=280	Hyponatremia Group n=66	Normonatremia Group n=214	p-value
Age, years	67.6±10.5	67±11	68±10	0.820
Male, n (%)	202 (72.2)	47 (71.2)	155 (72.4)	0.847
Hypertension, n (%)	185 (66.1)	41 (62.1)	144 (67.3)	0.438
Diabetes mellitus, n (%)	96 (34.3)	32 (48.5)	64 (29.9)	0.005
Coronary artery disease, n (%)	195 (69.6)	44 (66.7)	151 (70.6)	0.548
Atrial fibrillation, n (%)	124 (44.3)	39 (59.1)	85 (39.7)	0.020
NYHA class I-II n (%)	176 (62.9)	45 (68.2)	131 (61.2)	0.306
NYHA class I-II +AF, n (%)		23/45 (51.1)^a^	42/131 (32.1)^c^	0.022
NYHA class III-IV, n (%)	104 (37.1)	21 (31.8)	83 (38.8)	0.306
NYHA class III- IV +AF, n (%)		15/21 (71.4)^b^	44/83 (53%)^d^	0.028
Disease duration (years)	5.5 (3–12)	5.1 (4–11)	5.4 (3–9)	0.546
Resting heart rate (bpm)	82.5 ±19	82±12	84±19	0.215
**Laboratory measurements**				
Fasting glucose (mg/dL)	125±55	136±61	121±52	0.041
Creatinine (mg/dL)	124±0.3	1.25±0.30	1.24±0.3	0.662
LDL cholesterol (mg/dL)	103±42	106±49	102±40	0.461
Uric acid (mg/dL)	7.4±2.4	7.6±2.4	7.3±2.3	0.294
Sodium (mEq/L)	138±3.6	132±3.7	140±2.7	<0.001
Potassium (mEq/L)	4.4±0.5	4.5±0.5	4.3±0.5	0.513
Hs-CRP (mg/dL)	3.8 (1.5–7.3)	4.2 (1.8–6.7)	3.6 (1.2–7.8)	0.367
NT-proBNP, pg/mL	2605 (903–6825)	2916 (1170–9566)	2378 (867–6015)	0.199
Osmolality (mOsm/kg)	291±9	283±9	294±7	<0.001
Hemoglobin (g/dL)	12.8±2	12.5±1.7	12.9±1.9	0.393
**Echocardiographic parameters**				
LA diameter (mm)	46±7	45±6	46±7	0.546
LV diastolic diameter (mm)	59±7	59±7	60±8	0.634
Ejection fraction (%)	30±4	29±4	31±4	0.518
sPAP (mmHg)	42±14	40±13	42±14	0.343
Medications				
ACEI/ARB, n (%)	184 (65.7)	38 (57.6)	146 (68.2)	0.070
MRA, n (%)	157 (56.1)	44 (66.7)	113 (52.8)	0.021
Diuretic, n (%)	208 (74.3)	48 (72.7)	160 (74.8)	0.194
Betablockers, n (%)	236 (84.3)	54 (81.8)	182 (85)	0.183
Digoxin, n (%)	56 (20)	19 (28.8)	37 (17.3)	0.022

*ACEI: Angiotensinogen-converting enzyme inhibitor; ARB: Angiotensin receptor blocker; AF: Atrial fibrillation; Hs-CRP: High-sensitivity C-reactive protein; LA: Left atrium; LDL: Low-density lipoprotein; LV: Left ventricle; mOsm/kg: Milliosmoles per kilogram, MRA: Mineralocorticoid receptor antagonist; NT-proBNP: N terminal pro B-type natriuretic peptide; sPAP: Systolic pulmonary artery pressure. Between a and b, p=0120; between c and d, p=0.002.*

The hyponatremia group had a higher ratio of AF and DM than the normonatremia group. The ratios of patients with hypertension, CAD, diabetes mellitus, and NYHA functional class III–IV were similar within the two groups. Fasting glucose, rates of mineralocorticoid receptor antagonist (MRA), and digoxin use were higher in the hyponatremia group compared to the normonatremia group. Osmolality was lower in the hyponatremia group, as naturally expected. Age, gender, disease duration, resting heart rate, creatinine, LDL cholesterol, uric acid, potassium, hs-CRP, NT-proBNP, hemoglobin, LA and LV diastolic diameter, EF (%), and sPAP values were similar in the two groups. Patients with AF had lower sodium levels compared to those without AF (136±4.3 vs. 138±3.0 mEg/L, p=0.001) (Figure 1A) ( Table 1 ).

**Figure 1:** – A) Shows the comparison of sodium levels between the patients who have heart failure with normal sinus and atrial fibrillation. B) Demonstrates the ROC analysis that shows a poor diagnostic sensitivity and specificity of sodium levels to predict the possibility.

In patients with hyponatremia, the rates of AF were found to be significantly higher in patients with higher NYHA functional classes. Although there was no difference in terms of AF rates between NYHA class I–II and III–IV in patients with hyponatremia and HFrEF, AF rates showed statistically significant difference in patients with normonatremia and HF ( Table 1 ).

The results of univariate and multivariate logistic regression analysis to show the independent predictors of AF revealed advanced age, resting heart rate, and LA diameter. Diuretic and digoxin usage were found to be strongly correlated with the presence of AF ( Table 2 ).

**Table 2:** – Univariate and multivariate logistic regression analyses for the presence of atrial fibrillation

		Odds ratio	95% CI	p-value
**Univariate analyses**				
Age	0.076±0.023	1.079	1.031–1.170	0.001
Male gender	0.652±0.270	1.919	1.131–3.256	0.016
Hypertension	0.336±0.432	1.399	0.600–3.260	0.437
Diabetes mellitus	0.246±0.487	1.279	0.492–3.325	0.613
Coronary artery disease	-0.805±0.451	0.447	0.185–1.081	0.074
Functional capacity	0.026±0.419	1.027	0.451–2.334	0.950
Disease duration	1.196±0.576	0.827	0.271–2.493	0.729
Resting heart rate	0.041±0.008	1.042	1.026–1.059	<0.001
Fasting glucose	0.001±0.005	1.001	0.990–1.011	0.924
Creatinine	-0.025±0.182	0.976	0.682–1.395	0.892
LDL cholesterol	-0.007±0.003	0.993	0.987–0.999	0.024
Uric acid	0.172±0.100	1.188	0.976–1.446	0.086
Sodium levels	0.022±0.134	1.022	0.785–1.330	0.871
Potassium	-0.727±0.391	0.483	0.225–1.039	0.063
Hs-CRP	-0.012±0.016	0.988	0.958–1.020	0.461
NT-proBNP	0.001±0.001	1.000	0.999–1.001	0.071
Osmolality	-0.065±0.060	0.937	0.834–1.054	0.279
Hemoglobin	-0.174±0.131	0.840	0.650–1.086	0.183
LA diameter	0.046±0.013	1.047	1.021–1.516	<0.001
ACEI/ARB	-0.047±0.288	0.954	0.543–1.677	0.870
MRA	-0.163±0.290	0.850	0.481–1.501	0.575
Diuretic	1.448±0.364	4.256	2.086–8.685	<0.001
Beta-blockers	-0.165±0.388	0.848	0.396–1.814	0.671
Digoxin	1.876±0.365	6.526	3.193–13.340	<0.001
**Multivariate analysis**				
Age	0.045±0.015	1.046	1.016–1.177	0.003
Coronary artery disease	-0.805±0.451	2.058	1.122–3.777	0.020
Resting heart rate	0.041±0.009	1.041	1.023–1.060	<0.001
LA diameter	0.044±0.017	1.049	1.011–1.616	<0.001

*ACEI: Angiotensinogen-converting enzyme inhibitor; ARB: Angiotensin receptor blocker; Hs-CRP: High-sensitivity C-reactive protein; LA: Left atrium; LDL: Low-density lipoprotein; NT-proBNP: N terminal pro B-type natriuretic peptide; MRA: Mineralocorticoid receptor antagonist.*

The ROC analysis (AUC=0.458, 95% CI=0.397–0.527) revealed that blood sodium levels ≤135 mEq/L have poor diagnostic sensitivity (55%) and specificity (41%) for predicting AF. If the cut-off value of sodium level was adjusted to ≤130 mEq/L, higher sensitivity (70%) and poor specificity (31%) values were found (Figure 1B).

## Discussion

We report that the prevalence of AF was higher in outpatients with HFrEF and hyponatremia than in those with HFrEF and normonatremia, irrespective of the plasma osmolality levels and other confounding factors. There have been two studies in the literature showing higher rates of AF in patients with HFrEF and hyponatremia,^5 , 11^ which our results were concordant with. However, hyponatremia was not an inciting factor for the development of AF in the study. Hyponatremia is mild-moderate sensitive, but not specific for predicting the development of AF. Namely, AF is not present in every patient with hyponatremia and HFrEF.

The important predisposing and determining factors for AF development were advanced age, presence of CAD, increased resting heart rate and LA dimension, which were established as predictors of AF by previous studies.^9 , 10 , 15^ We reported a higher AF rate in patients with hyponatremia, irrespective of their NHYA functional class, as documented previously.^5 , 11^ Therefore, the coexistence of hyponatremia and AF may demonstrate HF severity. The rates of AF in the patients with normonatremia and HF were higher in NHYA class III–IV, which also means that the presence of NYHA III–IV status is an important reason for diuretic usage. Therefore, hyponatremia appears to be only a bystander variable.

The most common reasons for hyponatremia in patients with HF are diuretic usage and neurohormonal response, including an autonomic imbalance in favor of the sympathetic nervous system or renin-angiotensin system (RAS) activation.^1 , 5 , 16^

Many factors have been responsible for the association of hyponatremia with an increased risk for AF. Heart failure reduces stroke volume and arterial filling, which results in stimulation of arterial baroreceptors, arginine vasopressin (AVP) release, and RAS activation. RAS activation leads to increased aldosterone and angiotensin II levels. Angiotensin II alerts the thirst center of the brain and stimulates AVP release. The subsequent increase levels of aldosterone, angiotensin II, sympathetic system, and AVP release induce reduced renal blood flow, enhanced water retention, and sodium reabsorption.^4 , 17^ As a result of these neurohormonal changes, hypervolemia and hyponatremia occur. Some studies have shown increased levels of renin, angiotensin II, aldosterone, epinephrine, norepinephrine, and dopamine in patients with HF and hyponatremia compared to those with HF and normonatremia.^16 - 19^

Hyponatremia may also be a predictor of higher neurohormonal activation that suggests HF severity.^4^ Diuretics, especially thiazides, often result in hyponatremia, which promotes water retention due to enhanced AVP activation in the distal tubules.^20 , 21^ Hypervolemia leads not only to hyponatremia but also to atrial myocardial stretch, cardiac chamber, and pulmonary vein dilatation.^22^ Hyponatremia, theoretically, may also contribute to the development of AF, causing electrophysiological changes in the myocyte action potential.^23^ However, in clinical practice, it appears not to be a determinant of AF.

AF-induced rapid heart rate deleteriously affects left ventricle function, facilitates tachycardia, and predisposes apoptosis and myocardial fibrosis. Irrespective of the presence of HF, irregular heart rate and loss of atrial contraction results in a significant 7–9% and 20% reduction in cardiac output, respectively.^24^ When HF and AF co-exist, two intertwined entities make cardiac output decrease synergistically, and mortality increases.^8^ There is a common cause-effect relationship between these two entities.

Hyponatremia is frequently seen in patients with acute decompensated HF due to high diuretic usage and high sympathetic tonus triggering RAS activation.^5 , 21^ Our findings are not concordant with that of the study of Cavusoglu et al. showing hyponatremia with a prevalence of 24%, and AF with a prevalence of 33%.^11^ Bavishi et al. found that the prevalence of hyponatremia and AF in outpatients with HFrEF was 14.8% and 37.6%, respectively.^5^ Our study has presented a hyponatremia rate of 23.5%, but a higher AF rate of 44.3%, because we performed ambulatory Holter ECG to find the presence of paroxysmal or persistent AF. AF rates are higher than we expected in ambulatory 24-hour Holter ECG monitoring in patients with HF.^2 , 15^

### Study limitations

We presented missing data related to diuretic doses and albumin levels, which could affect the sodium levels.

## Conclusion

Current findings yield insights on the pathogenesis of AF in patients with established HF. Although hyponatremia plays a key role in the deterioration of HF status, we found that low serum sodium concentration ≤135 mEq/L is not related to the probability of AF.
